# Telomerase Reverse Transcriptase Polymorphism rs2736100: A Balancing Act between Cancer and Non-Cancer Disease, a Meta-Analysis

**DOI:** 10.3389/fmed.2018.00041

**Published:** 2018-02-27

**Authors:** Reinier Snetselaar, Matthijs F. M. van Oosterhout, Jan C. Grutters, Coline H. M. van Moorsel

**Affiliations:** ^1^Interstitial Lung Diseases Center of Excellence, Department of Pulmonology, St Antonius Hospital, Nieuwegein, Netherlands; ^2^Interstitial Lung Diseases Center of Excellence, Department of Pathology, St Antonius Hospital, Nieuwegein, Netherlands; ^3^Division of Heart and Lung, University Medical Center Utrecht, Utrecht, Netherlands

**Keywords:** telomerase, telomere, cancer, single nucleotide polymorphism, degenerative disease

## Abstract

The enzyme telomerase reverse transcriptase (TERT) is essential for telomere maintenance. In replicating cells, maintenance of telomere length is important for the preservation of vital genetic information and prevention of genomic instability. A common genetic variant in *TERT*, rs2736100 C/A, is associated with both telomere length and multiple diseases. Carriage of the C allele is associated with longer telomere length, while carriage of the A allele is associated with shorter telomere length. Furthermore, some diseases have a positive association with the C and some with the A allele. In this study, meta-analyses were performed for two groups of diseases, cancerous diseases, e.g., lung cancer and non-cancerous diseases, e.g., pulmonary fibrosis, using data from genome-wide association studies and case-control studies. In the meta-analysis it was found that cancer positively associated with the C allele (pooled OR 1.16 [95% CI 1.09–1.23]) and non-cancerous diseases negatively associated with the C allele (pooled OR 0.81 [95% CI 0.65–0.99]). This observation illustrates that the ambiguous role of telomere maintenance in disease hinges, at least in part, on a single locus in telomerase genes. The dual role of this single nucleotide polymorphism also emphasizes that therapeutic agents aimed at influencing telomere maintenance should be used with caution.

## Introduction

Telomere biology is emerging as a significant factor in an increasing number of diseases (1–4). Studies have found disease associations with both abnormal telomere length and with genetic variants are related to telomere biology (5–7). Telomeres are non-coding tandem repeats spatially organized by specialized proteins that maintain stability of the chromosome ends (8–10). Furthermore, telomeres serve as a buffer against the shortening of chromosomes, thereby preventing the loss of vital genetic information (11). To maintain replicative potential, telomeres can be elongated by the ribonucleoprotein telomerase (12, 13). Telomerase consists of a catalytic protein component, encoded by the gene telomerase reverse transcriptase (*TERT*), and a RNA template, encoded by telomerase RNA component.

Over the course of the human lifespan, the average length of telomeres can be disproportionally influenced by a number of factors, leading to a broad spectrum of diseases. Genetic variation in telomere maintenance genes has been shown to either accelerate or prohibit telomere shortening. Genomic mutations in the coding regions of telomerase genes are primarily found in degenerative diseases like dyskeratosis congenita, aplastic anemia, and idiopathic pulmonary fibrosis (IPF) (14–16). These mutations generally lead to a decrease in telomerase activity (17) and shorter telomeres in mutation carriers who develop fatal disease due to organ failure. Mutations in the coding regions of telomerase genes are very rare in cancer, however, somatic mutations in the promotor region of the *TERT* gene has been reported in the context of several cancer types (18–20). These mutations generally lead to an increase in telomerase activity, which corroborates the observation of high levels of telomerase activity in cancer cells (21). Telomerase activation and the subsequent telomere elongation lead to the immortalization of cells and prevent fatal instability of the chromosomes, opening up the possibility of unrestricted cell proliferation (21, 22).

However, Telomere biology is an ambiguous factor in cancer pathology (23–25). In healthy individuals it is thought that the restricted transcription of telomerase and the resulting limited number of cell divisions present a barrier to unlimited replication of somatic cells, thus preventing cancer (26, 27). However, other research suggests that telomere shortening can lead to chromosomal instability in the form of chromosome fusion, genomic copy addition, deletion, and mutation, which in turn can lead to tumor initiation (22, 28–31). This duplicity is apparent in humans, where both long and short telomere length of white blood cells has been associated with different cancers (32–36). As short telomeres can lead to damaged chromosomes, it is proposed that long telomeres postpone senescence, thereby increasing the risk for cells to acquire genetic abnormalities that facilitate tumorigenesis (35, 37, 38).

Besides rare mutations in telomerase genes, common genetic variation in these genes has also been associated with disease. A well-studied example is the single nucleotide polymorphism (SNP) rs2736100 in the *TERT* gene (5p15.33). Interestingly, the first report on a disease association to this SNP, was to the non-cancerous disease IPF (39). An IPF susceptibility odds ratio (OR) of 1.82 [95% CI: 1.47–2.22] was found for the A allele of this SNP. The second report on this SNP showed an association between lung cancer and the C allele of this SNP (40). Later studies have shown an association between the A allele and shorter blood cell telomere length, while it follows that the C allele is associated with longer telomeres (41, 42). This duality in disease association of the rs2736100 alleles might reflect a fundamentally different role of telomere biology in cancerous diseases as opposed to non-cancerous diseases. Such a dichotomy would underline that therapeutic agents influencing telomere length or telomerase activity should be used with caution, as both (too) long and short telomeres could lead to disease.

The aim of this study is to conduct a systematic review and meta-analysis of disease association studies with *TERT* SNP rs2736100 and to gain insight in the balancing act between telomere maintenance and disease predisposition.

## Materials and Methods

### Study Selection

**Figure 1:**
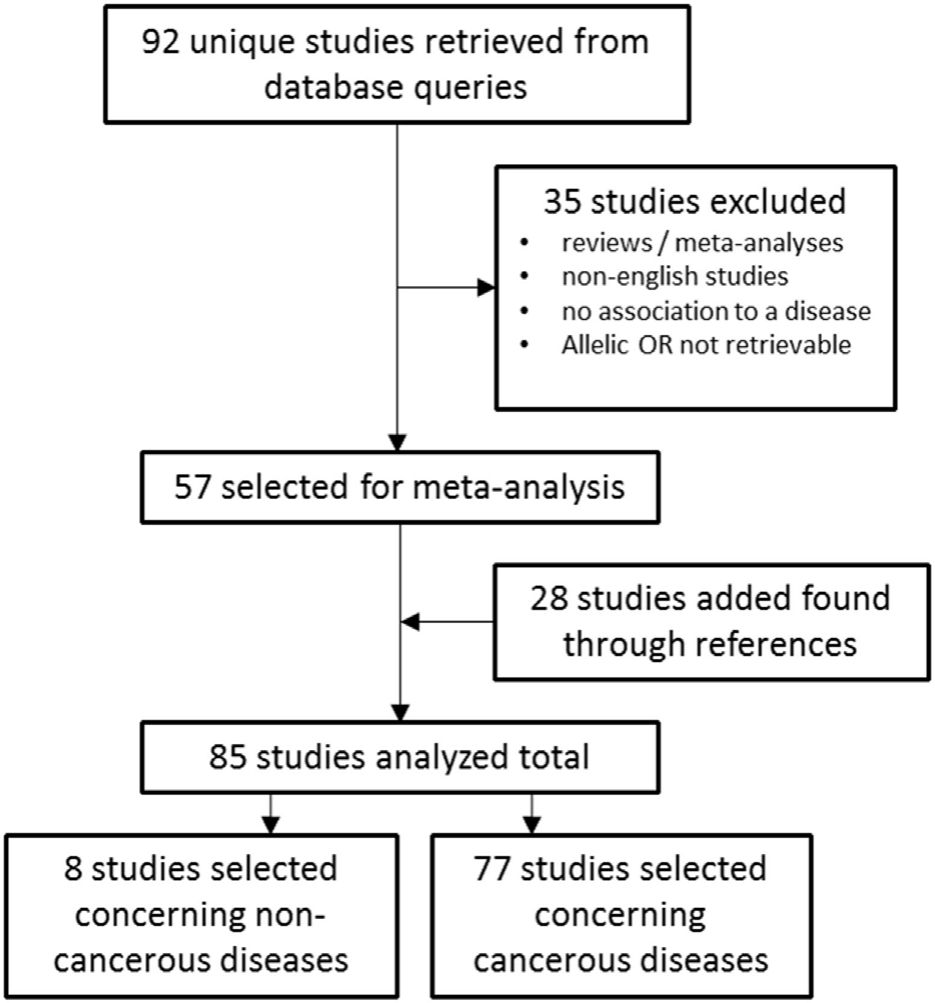
Flowchart of studies included in the meta-analysis.

The electronic databases PubMed^1^ and Embase^2^ were queried for studies on *TERT* SNP rs2736100 by using “rs2736100” as search input (Figure 1). Initially 92 studies were found. After selecting for papers pertaining to the subject of these review 57 studies remained. Of these, 49 studies described associations between *TERT* SNP rs2736100 and cancer, and 8 studies described associations between this SNP and non-cancer disease. Another 28 studies were added through references found in the original studies. Excluded were reviews and meta-analyses, and studies from which no OR data was available or could be calculated. Furthermore, studies were excluded that did not provide definite data on which allele was associated with risk for the investigated disease. Finally, 85 studies were included of which 77 described association with cancer and 8 with non-cancer and the *TERT* SNP, respectively.

### Eligibility Criteria

Included in this meta-analysis were case-control and genome-wide association studies assessing the association between *TERT* SNP rs2736100 and disease. These studies were furthermore included when the associated allele and the used inheritance model were clearly derivable from the study. Results of meta-analyses were excluded; however, these studies were searched for eligible studies to be included in the present study. This lead to further inclusion of studies that did not find significant associations between rs2736100 and disease, thereby preventing major publication bias. Finally, systematic reviews, abstracts, non-english studies and studies investigating rs2736100 not in the context of disease were excluded from the meta-analysis.

### Data Extraction

The following data was derived from each study: first author, year of publication, number of cases and controls, associated disease (cancer and non-cancer), and OR and 95% confidence interval (95% CI). Furthermore, the used inheritance model was checked as well as in which allele specifically was associated with the studied disease. When this information was not provided it was derived from the provided genotype data when possible.

### Bias Evaluation

(Publication) bias was evaluated by visual inspection of Doi plots, as well as calculates the Luis Furuya-Kanamori (LFK) index, which provides a statistic for the amount of bias in a Doi plot (No asymmetry: LFK index within ±1, minor asymmetry: LFK index exceeds ±1 but within ±2, major asymmetry: LFK index exceeds ±2).

### Statistical Analysis

rs2736100 associations and (publication) bias were both analyzed using meta-analysis software MetaXL 4.0 (EpiGear International, Sunrise Beach, Australia). Due to the large number of different diseases that were included in this study, a high level of variation in study outcome, heterogeneity, was expected. The meta-analysis was, therefore, performed using the inverse variance heterogeneity method, to determine the pooled result and heterogeneity (43). Many studies do not provide genotype data. Therefore, we performed meta-analysis on the OR and 95% CI for allelic association.

## Results

### Non-Cancerous Diseases

**Figure 2:**
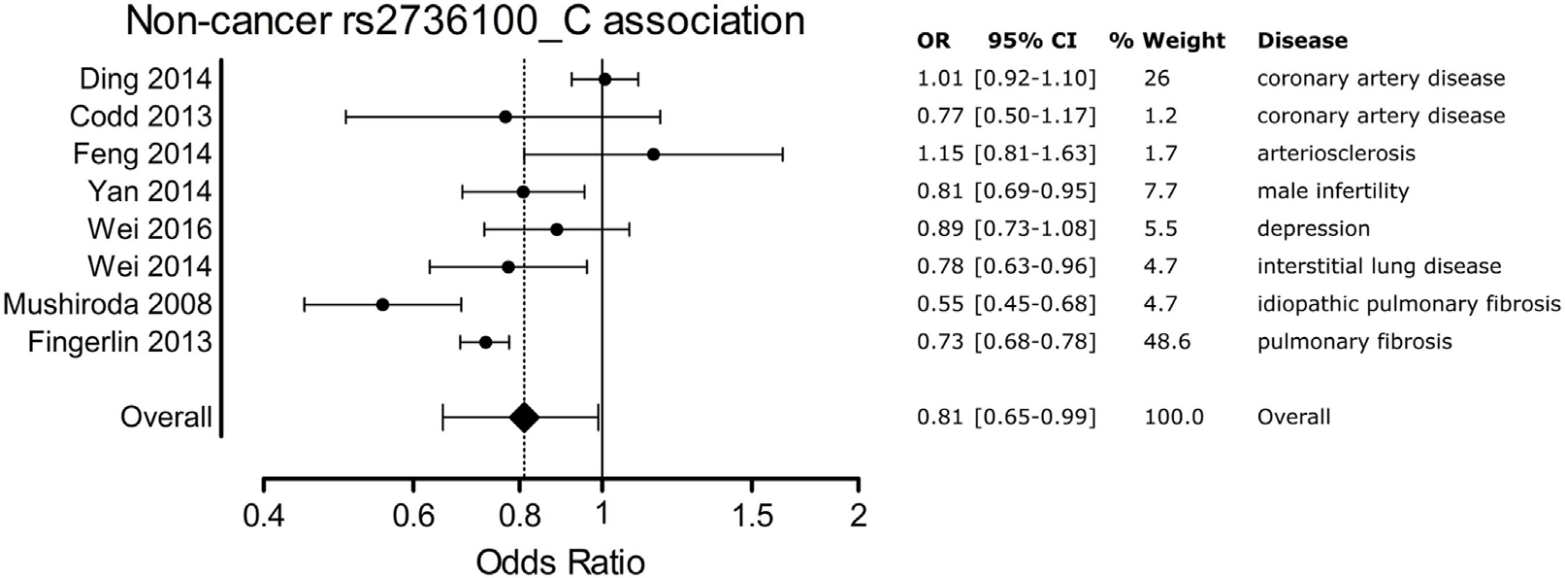
Meta-analysis of non-cancer association to rs2736100 allele C. OR, odds ratio; 95% CI, 95% confidence interval.

A meta-analysis was performed to analyze the association of *TERT* SNP rs2736100 with a group of cancer diseases and with a group of non-cancerous diseases. Table 1 shows all included studies. For the non-cancer group, 8 studies were found with diagnoses of pulmonary fibrosis and coronary heart disease among others. Each of these eight studies showed either a negative association between the C allele and disease or a non-significant result. Figure 2 shows a forest plot of a meta-analysis of these studies using the inverse variance heterogeneity model (43). The OR in this figure is an effect measure for the association with the C allele of rs2736100 in a co-dominant model. The pooled negative association with disease for the C allele was significant with an OR ratio of 0.81 [95% CI: 0.65–0.99]. Presence of the C allele is protective for non-cancerous diseases.

**Table 1:** Studies included in the meta-analyses.

			Association rs2736100_C		
Study	Cases	Controls	Odds ratio	95% CI	Disease
(44)	245	489	1.12	0.90–1.39	Pediatric brain tumor
(45)	445	497	1.29	1.07–1.55	Lung cancer
(46)	1,154	1,137	1.24	1.10–1.39	Lung cancer
(47)	1,896	1,939	1.13	1.03–1.24	Pancreatic cancer
(48)	1,094	1,100	1.14	1.01–1.30	Lung cancer
(49)	4,441	5,194	1.22	1.15–1.29	Lung cancer
(50)	5,550	7,585	0.91	0.99–0.84	Pancreatic cancer
(51)	976	1,057	1.26	1.11–1.43	Glioma
(52)	196	229	1.65	1.17–2.32	Lung cancer
(53)	13,265	40,245	1.08	1.12–1.04	Endometrial cancer
(54)	243	246	1.20	0.92–1.55	Gastric cancer
(55)	386	587	0.94	0.78–1.14	Liver cancer
(41)	22,233	64,762	0.77	0.50–1.17	Coronary artery disease
(56)	663	420	0.99	0.79–1.25	Renal cell carcinoma
(57)	845	1,190	1.27	1.12–1.44	Glioma
(58)	1,514	2,470	1.01	0.92–1.10	Coronary artery disease
(59)	1,136	1,012	1.02	0.90–1.15	Gastric cancer
(60)	639	649	1.37	1.18–1.61	Glioma
(61)	3,131	3,702	1.02	0.94–1.11	Skin cancer
(62)	84	257	1.15	0.81–1.63	Arteriosclerosis
(63)	2,477	6,550	0.73	0.68–0.78	Pulmonary fibrosis
(64)	970	525	1.17	1.04–1.33	Bladder cancer
(65)	510	913	1.17	1.00–1.37	Skin cancer
(66)	3,264	1,793	0.98	0.77–1.25	Colorectal cancer
(67)	2,308	2,321	1.48	1.36–1.62	Lung cancer
(68)	8,559	9,378	1.25	1.20–1.31	Lung cancer
(69)	1,145	1,142	0.96	0.78–1.17	Breast cancer
(70)	716	716	1.17	1.00–1.38	Lung cancer
(71)	717	202	1.57	1.25–1.96	Myeloproliferative neoplasms
(72)	104	135	1.01	0.70–1.46	Colorectal cancer
(73)	855	844	1.16	1.02–1.33	Lung cancer
(74)	1,212	1,339	1.14	1.02–2.27	Lung cancer
(75)	349	914	0.81	0.68–0.97	Testicular cancer
(76)	16,039	16,430	0.93	0.91–0.96	Colorectal cancer
(77)	370	1,173	1.38	1.23–1.56	Lung cancer
(78)	584	400	1.77	1.47–2.12	Myeloproliferative neoplasms
(79)	518	1,201	1.64	1.42–1.91	Glioma
(80)	855	1,160	1.16	1.01–1.33	Glioma
(81)	4,543	5,505	1.38	1.31–1.47	Lung cancer
(82)	193	197	1.29	1.00–1.67	Lung cancer
(83)	5,739	5,848	1.09	1.03–1.15	Lung cancer
(84)	370	1,263	1.10	0.93–1.29	Colorectal cancer
(85)	2,283	2,785	1.18	1.09–1.27	Lung cancer
(86)	304	319	1.33	1.06–1.67	Lung cancer
(87)	690	1,538	1.19	1.03–1.38	Bladder cancer
(88)	5,457	4,493	1.38	1.30–1.47	Lung cancer
(40)	5,870	9,319	1.14	1.08–1.20	Lung cancer
(89)	2,086	11,034	1.27	1.19–1.37	Lung cancer
(90)	226	806	1.22	0.99–1.51	Acute myeloid leukemia
(91)	226	806	1.44	1.10–1.88	Glioma
(39)	242	1,496	0.55	0.45–0.68	Idiopathic pulmonary fibrosis
(92)	352	447	1.18	0.97–1.45	Lung cancer
(34)	277	831	1.19	1.04–1.37	Skin cancer
(93)	1,681	1,635	1.16	1.04–1.30	Lung cancer
(94)	3,534	4,098	1.08	1.02–1.16	Breast cancer
(95)	1,955	1,995	1.11	1.00–1.23	Pancreatic cancer
(96)	596	1,480	1.08	0.94–1.23	Endometrial cancer
(97)	1,854	4,949	1.30	1.19–1.41	Glioma
(98)	810	3,080	1.23	1.10–1.37	Glioma
(99)	1,029	1,668	1.31	1.17–1.47	Glioma
(100)	660	523	0.92	0.78–1.09	Breast cancer
(100)	372	363	0.95	0.77–1.16	Prostate cancer
(101)	569	656	1.23	1.05–1.45	Acute lymphoblastic leukemia
(102)	1,878	3,670	1.27	1.19–1.37	Glioma
(103)	5,992	13,531	1.34	1.28–1.41	Lung cancer
(104)	807	708	1.24	1.05–1.48	Lung cancer
(105)	810	4,479	1.31	1.16–1.48	Myeloproliferative neoplasms
(106)	719	6,030	1.08	0.97–1.21	Lung cancer
(107)	10,812	13,913	1.15	1.10–1.20	Lung cancer
(108)	661	1,347	1.32	1.14–1.52	Lung cancer
(109)	1,045	8,403	0.75	0.67–0.85	Testicular cancer
(110)	1,660	1,299	1.39	1.28–1.50	Glioma
(111)	1,618	7,736	1.39	1.28–1.50	Glioma
(112)	239	553	1.30	1.04–1.61	Lung cancer
(113)	1,033	1,053	1.16	1.03–1.32	Cervical cancer
(114)	1,552	1,605	1.20	1.09–1.33	Lung cancer
(115)	370	686	0.78	0.63–0.96	Interstitial lung disease
(116)	1,404	5,040	1.24	1.10–1.39	Lung cancer
(117)	239	1,197	0.89	0.73–1.08	Depression
(118)	692	3,992	1.51	1.35–1.71	Glioma
(119)	580	580	0.81	0.69–0.95	Male infertility
(120)	1,735	1,036	1.11	0.99–1.24	Lung cancer
(121)	524	524	1.34	1.13–1.60	Lung cancer
(122)	1,425	3,011	1.26	1.11–1.43	Lung cancer
(123)	784	782	1.28	1.11–1.47	Lung cancer
(124)	2,096	2,147	1.38	1.27–1.51	Esophageal cancer

### Association with Cancer

**Figure 3:**
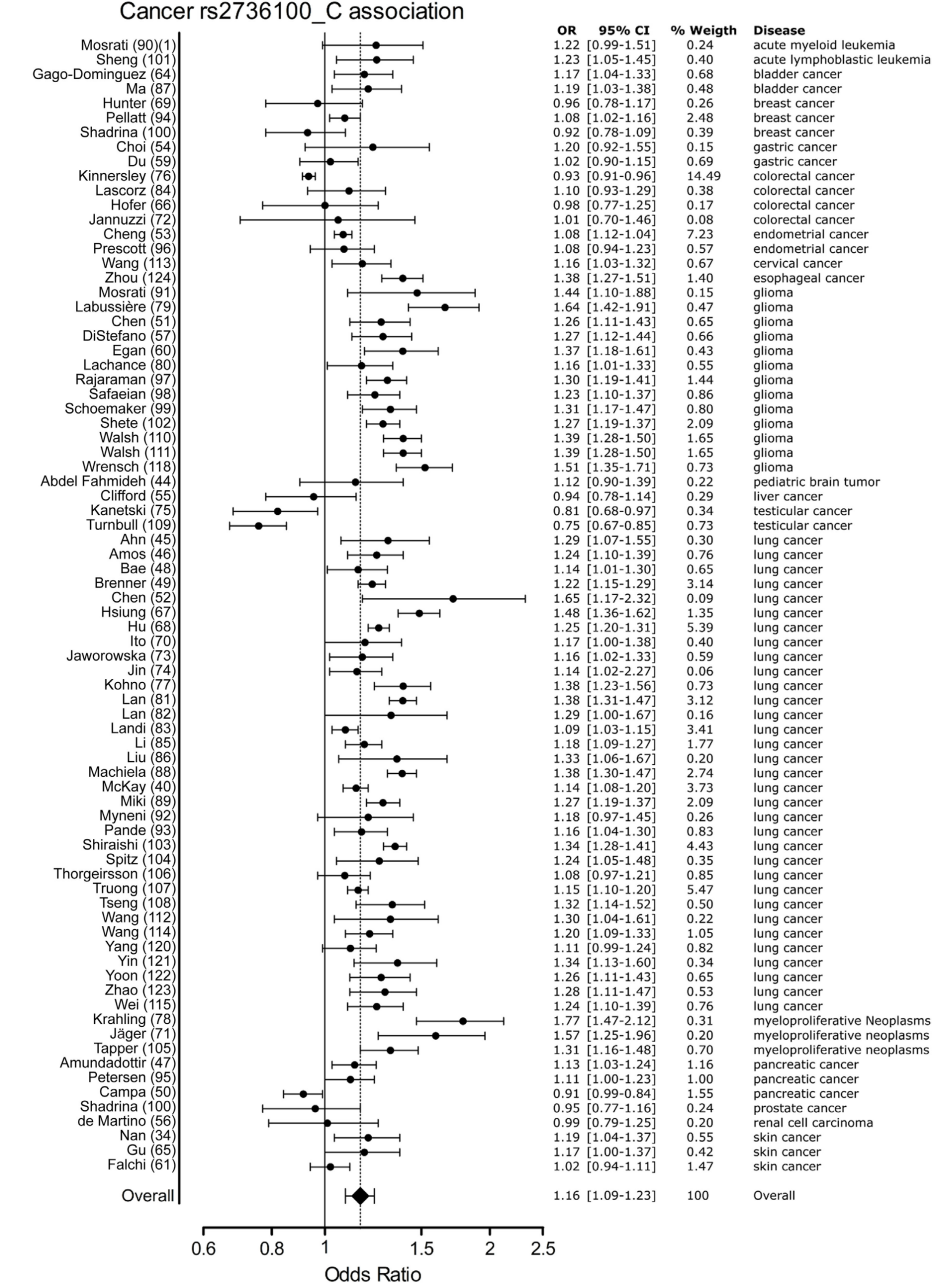
Meta-analysis of cancer association to rs2736100 allele C. OR, odds ratio; 95% CI, 95% confidence interval.

Meta-analysis for associations with cancer included 77 studies. These studies included a variety of cancers of which the majority (*n* = 46) involved studies on lung cancer (*n* = 33) and glioma (*n* = 13). The majority of studies reported a positive association with the C allele of rs2736100. However, four of the included studies reported a negative association with the C allele, these included two studies on testicular cancer, one on colorectal cancer and one on pancreatic cancer (50, 76, 109). In the meta-analysis of cancer studies, the pooled effect size was significant with a pooled OR of 1.16 [95% CI: 1.09–1.23] (Figure 3) and shows that the C allele is a risk allele for cancer.

**Figure 4:**
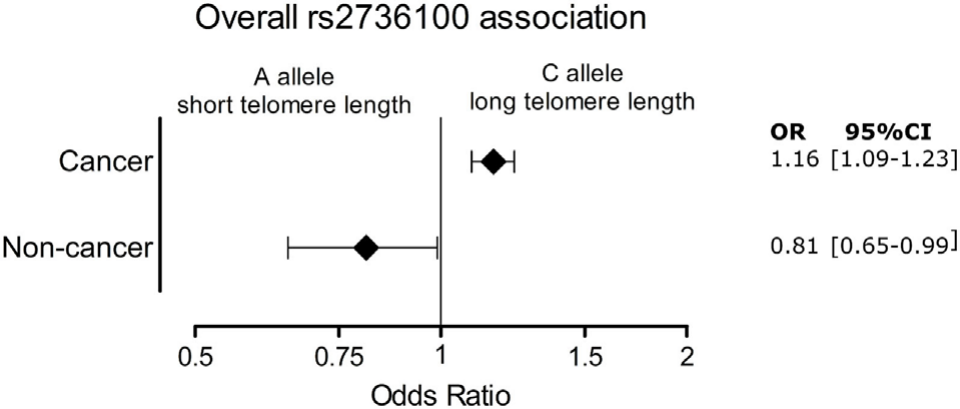
Overall association of the cancer and age-related group of diseases with telomerase reverse transcriptase rs2736100 allele C. OR, odds ratio; 95% CI, 95% confidence interval.

Figure 4 shows the significant pooled ORs for the meta-analysis of cancer and non-cancer diseases.

**Figure 5:**
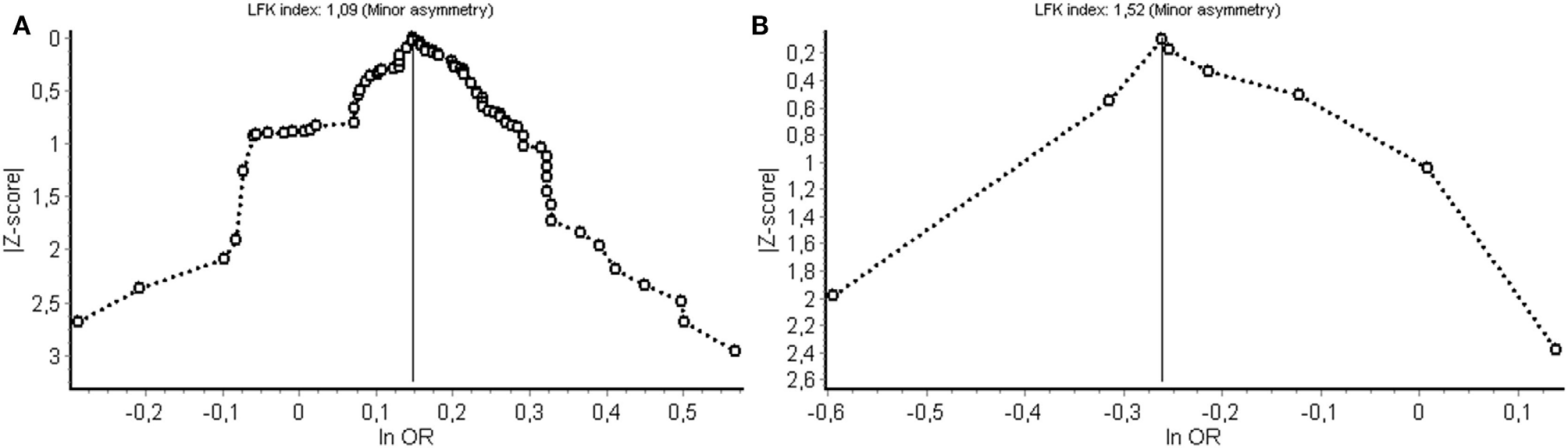
Evaluation of (publication) bias for the cancer **(A)** and non-cancer **(B)** meta-analysis. LFK index, Luis Furuya-Kanamori index; OR, odds ratio.

Potential bias from publication selection and other sources was evaluated using Doi plots and quantified by the LFK index (Figure 5). Figure 5A shows the Doi plot for the cancer meta-analysis. A LFK index of 109 was found from indication minor influence from publication bias or bias from other sources. Figure 5B shows the Doi plot for non-cancer diseases.

## Discussion

The results of the meta-analyses illustrate the duality of telomere biology in disease predisposition. Pooled analysis showed that non-cancerous diseases, such as pulmonary fibrosis and coronary artery disease, associate positively with the telomerase A allele that is linked to shorter telomeres. Pooled analysis of cancer studies, however, showed an association with the opposite allele, the C allele that is linked to longer telomere length. This is supported by a recent study that showed an association of genetically increased telomere length and cancer, while the opposite protected against non-cancerous diseases (125). This two-sided association suggests opposite roles of telomere length in cancerous and non-cancerous diseases.

The *TERT* SNP rs2736100 has robustly been associated with telomere length in healthy controls (41, 42). Studies showed that presence of the A allele is associated with shorter telomere length. This is in congruence with observations made in IPF, cardiovascular disease, and male infertility, where patients have been shown to have relatively short leukocyte telomeres (126–128). This SNP could, therefore, be part of the genetic background that increases susceptibility to these diseases in combination with other cellular and environmental factors that might cause increased cellular turnover (129). On the other hand, the C allele, which is associated with most cancer types, is associated with longer telomere length in health (42, 101). How the rs2736100 SNP influences telomere length is presently not understood. The SNP is located in intron 2 in *TERT* and was suggested to influence telomerase activity or to be in strong linkage with a functional variant in *TERT* (83, 101).

The dual associations of the SNP in this study would support the hypothesis that telomere maintenance is at an intersection between cancer- and premature-aging (130, 131). Cancer and aging share many molecular pathways, including telomere maintenance pathways. And while aging is associated with a progressive decrease in telomere length, cancer is characterized by immortalization of the cell often through telomerase activation (21, 132). Short telomeres accelerate aging through cell senescence, but long telomeres postpone senescence, which in turn, facilitates survival of cells with acquired oncogenic DNA alterations and thereby promotes tumorigenesis (35, 37, 38). The meta-analysis of cancer studies showed a pooled positive correlation with the *TERT* allele that is known to cause longer telomeres. This suggests that the majority of patients develop cancer due to a.o. the presence of long telomeres in tumor initiating cells. On the other hand, it is also well understood that critically short telomeres lead to chromosomal instability, which can cause tumorigenesis (32, 33, 36). Although the results of the meta-analysis suggest this if not the cause in the majority of cases, four cancer studies were included that originally reported an association with the *TERT* allele for short telomeres. For colorectal cancer there is general agreement that short telomere length increases tumor initiation by causing chromosomal instability (133). This would account for the association of colorectal cancer with the A allele of rs2736100 that is also associated with short telomere length (76). Testicular cancer is also associated with the A allele (109). Telomerase activity is restricted in most tissues, one exception being germ cells (21). It is assumed that in highly proliferative tissues, a genetic factor decreasing telomerase activity may cause chromosomal instability leading to cancer.

Most non-cancer diseases showed an association with the A allele of rs2736100 SNP. Regarding telomere biology, disease susceptibility for pulmonary fibrosis is opposite to that of lung cancer, while both diseases are highly associated with smoking behavior. In case of short telomeres, smoking may cause increased senescence with subsequent pulmonary fibrosis, while in case of long telomeres; smoking may cause DNA damage in cells with sustained proliferative capacity. For coronary artery disease no significant allelic association has been found for rs2736100. But reports have shown a significant effect of this variant when analyzed in combination with other risk loci or when analyzed in a dominant model (41, 58, 62). However, conflicting data were found for the direction of the association, which could have been due to the ethnic background of the population. Codd et al. reported an association between coronary artery disease and the A allele in a Caucasian cohort. Both Feng et al. and Ding et al. study Asian populations found no association or an association with the C allele (58, 62).

A limitation is that although an exhaustive literature search was performed, studies could have been overlooked. Some studies were excluded because of missing genotype data and some described an association without reporting the associated allele. Furthermore, it should be emphasized that the pathogenesis for most of the diseases mentioned in this study is not fully known and is suggested to be complex, involving both genetic and environmental factors. Another limitation is the discrepancy in the number of studies found for cancer vs non-cancer. This results in a strong influence of interstitial lung disease on the pooled OR. Finally, (publication) bias was evaluated for both the cancer and non-cancer study (Figure 5). For both, meta-analyses asymmetry was minor and the corresponding bias introduced by study selection and other sources is, therefore, also considered to be of minor influence on the pooled result.

The ambiguous effects of telomere maintenance do pose a great challenge for the development of therapeutic agents, for instance putative anti-aging therapeutics. Therapeutic agents aimed at increasing telomere length should be used with caution (25, 134). Telomerase activating agents used in the context of degenerative or aging-related diseases could facilitate tumorigenesis or lead to proliferation of untargeted tissues (131, 135). On the other hand, in relation to cancer, telomerase inhibiting agents are attractive candidates, but could lead to accelerated tissue degeneration or negatively affect stem cell function and immune response as this requires increased telomerase activity to sustain a high level of cell proliferation (135, 136). To develop disease-specific treatment, future research should be aimed at further understanding of optimal telomere length per specific tissues during life, and at the ability to target specific cell-types or tissues very accurately.

## Conclusion

Meta-analyses showed that the *TERT* SNP rs2736100 C allele is positively associated with multiple cancerous diseases, while the A allele is positively associated with predisposition to non-cancerous (degenerative) diseases. Because the SNP is known for its influence on telomere length, this result illustrates that optimal telomere maintenance balances between increasing the risk for cancer or for non-cancerous diseases. This underlines the caution that should be taken when developing therapies that influence telomere length.

## Author Contributions

RS and CM contributed to the study concept and design, data analysis and interpretation, writing of the manuscript and final approval; had full access to all the data in the study and takes responsibility for the integrity of the data and the accuracy of the data analysis. MO and JG contributed to the study concept and design, writing of the manuscript and final approval; had full access to all the data in the study and takes responsibility for the integrity of the data and the accuracy of the data analysis.

## Conflict of Interest Statement

The authors declare that the research was conducted in the absence of any commercial or financial relationships that could be construed as a potential conflict of interest.

## Footnotes

^1^http://www.ncbi.nlm.nih.gov/pubmed.

^2^https://www.embase.com/#quickSearch/default.
